# Attitude & belief towards mental illness and psychiatry as a faculty among medical students at International Medical University, Malaysia

**DOI:** 10.1192/bjo.2021.362

**Published:** 2021-06-18

**Authors:** Debakanta Behera, Ji Yen Ku

**Affiliations:** 1 NWBH NHS Foundation Trust; 2 5th year medical undergraduate, University of Liverpool

## Abstract

**Aims:**

Third year Medical students from the International Medical University, Malaysia were assessed regarding their commonly held attitudes and beliefs for the mental illness in general as well as with respect to psychiatry as a faculty through a survey monkey based survey,

**Background:**

Commonly held perceptions and prejudices often can be overcame by education and early exposure to facts which also holds true with medical students and their attitude as well as expectations to psychiatry. Ever growing awareness regarding the Mental illness has helped but is unable to complete address the stigma and prejudices associated with it. Also Early exposure to psychiatry in medical education can provide a positive experience to medical students including germinating an interest in psychiatry as a career choice among the students.

**Method:**

42 students of 3rd and 4th year medical school from International Medical University, Malaysia, some without any exposure to psychiatry, were participated in a survey created on a cloud based online survey link and responded to a questionnaire about the attitude and belief towards mental Illness as well as Psychiatry as a career choice. The results were analysed and data interpreted.

**Result:**

Most students (85%) though agreed that psychiatry is a rapidly expanding frontier of medicine sadly only 20% stated that it would be one of the top three career choice. Just under the 50% of the students stated that the psychiatric patients are more likely to harm others. About 95% felt that psychiatric consultations of patients with medical and surgical health problems would be helpful and 90% students shared that they would not feel embarrassed about someone from their family if diagnosed with mental illness.

**Conclusion:**

Psychiatric exposure in medical education has been recognised as inadequate in general and often exposing medical students to psychiatry early helps improving the stigma and prejudices associated with mental illness. It will also give them sufficient exposure to assess the illness holistically keeping mental health in mind while treating physically ill people and also may inspire them to choose psychiatry as a career choice in a rapidly developing and conservative country such as Malaysia where mental health services are largely inadequate and is the second biggest health issue.

